# Blood components are essential to regulate microcirculatory blood flow

**DOI:** 10.1186/s13054-017-1621-5

**Published:** 2017-03-08

**Authors:** Nicolas Morel, Marie Moisan

**Affiliations:** 1 Service de réanimation polyvalente, Polyclinique Jean Villar, Avenue Maryse Bastié, 33520 Bruges, France; 2 Pôle des urgences adultes, Hôpital Pellegrin CHU Bordeaux, Place Amélie Raba Léon, 33000 Bordeaux, France

We read with interest the article by Jacob and colleagues [1] on the regulation of microcirculatory blood flow. In our opinion, the authors missed an important parameter, i.e., blood components and, particularly, red blood cells (RBC). This is regrettable since blood composition is the main parameter that we can influence by our therapies (fluid resuscitation, blood or albumin transfusion) in shocked patients.

RBC may use different contradictory pathways to modulate microvascular flow by modifying nitric oxide (NO) bioavailability, which is responsible for precapillary dilatation and capillary perfusion.

On the one hand, erythrocytes may reduce NO bioavailability through hemoglobin NO scavenging; on the other hand, enhancing hematocrit may increase viscosity and wall shear stress (WSS), a crucial agonist enabling endothelium NO release.

Thus, in hypoxic conditions, RBC may sense tissue oxygen tension and release vasodilatory agents such as NO or ATP.

In moderate hemodilution, blood viscosity is reduced, cardiac output increases, wall shear rate (WSR; blood velocity) increases and WSS is unchanged (WSS = WSR × viscosity). NO bioavailability increases because of reduced NO scavenging by erythrocytes, leading to systemic vasoplegia. In extreme hemodilution [2] (hematocrit at 11%), viscosity, WSR, and WSS drop. Functional capillary density (FCD), which reflects microcirculatory flow, decreases. In this model, enhancing blood viscosity with high viscosity-plasma expander increased FCD. In the same way, in a murine model of hemorrhagic shock, transfusion of fresh RBC without oxygen-carrying capacity restored blood viscosity, and enhanced FCD, microvascular flow, and systemic hemodynamics. Recently, Tanaka and colleagues [3] observed that RBC transfusion improved sublingual FCD in humans in hemorrhagic shock with active blood loss.

We have known since 1999 from the results of a trial using modified human hemoglobin in traumatic hemorrhagic shock [4] that modifying blood properties could be harmful. Patients in this study had a better blood systolic pressure than control patients, but more of them died. Free hemoglobin scavenges NO, enhancing precapillary vasoconstriction and impairing capillary perfusion.

We do not share the authors’ opinion that it is “better to choose hemorrhagic rather than septic shock for yourself”. Guidelines for traumatic hemorrhagic shock management [5] do not consider RBC transfusion for their microcirculatory properties and viscosity improvement, but only for their oxygen-delivering capacity, which is altered for many hours after transfusion, when blood is stored. Recommending a low hemoglobin trigger delays RBC transfusion when microcirculation flow is already impaired.
