# Supplementary material for: Blood components are essential to regulate microcirculatory blood flow
Source: Crit Care. 2017 Mar 8;21:49. doi: 10.1186/s13054-017-1621-5 (PMC5341198; doi:10.1186/s13054-017-1621-5)
Supplement: Related texts

# Reply: Authors’ response

**Authors:** Matthias Jacob, Daniel Chappell, Bernhard F. Becker

Nicolas Morel and Marie Moisan have drawn attention to the special but confounding role played by RBC in modulating regional microcirculatory flow distribution. As they correctly point out, contributory effects include changes in blood viscosity and scavenging or provision of vasoactive metabolites. Such influences must be expected, especially in conjunction with transfusions of RBC in situations of treatment or prevention of shock [6]. Although the effect of blood transfusion on blood viscosity and flow resistance has fascinated physiologists and experimental clinicians for over 100 years, the action of RBC on blood flow distribution has not been resolved satisfactorily. This is, in part, due to the fact that microvascular flow resistance arises mainly from the leukocyte component of whole blood, and not from RBC-mediated changes in viscosity [7]. With respect to scavenging of endothelium-derived vasodilatory NO by the hemoglobin of RBC, such an effect may be countered by the recently described presence of an active type-3 NO synthase in RBC [8]. In addition, supplying oxygen to the tissue provokes the production of vasoconstrictors in vivo. This realization has disproved other hypotheses proposing oxygen-dependent destruction or removal of vasodilators by RBC [9].

These few examples of the problems still associated with understanding the mechanisms by which RBC may influence regional flow distribution illustrate the continued necessity of conducting well-planned clinical studies for the improvement of patient outcome in situations of shock. Although at first sight the approach to management of traumatic hemorrhagic shock seems more clear than that of septic states, Morel and Moisan have provided good reasons why this is not resolved.
